# Supplementary material for: Does behavior mediate the effect of weather on SARS-CoV-2 transmission? evidence from cell-phone data
Source: PLoS One. 2024 Jun 21;19(6):e0305323. doi: 10.1371/journal.pone.0305323 (PMC11192350; doi:10.1371/journal.pone.0305323)
Supplement: S1 Table — (DOCX) [file pone.0305323.s001.docx]

**Table S1. Detailed regression results between control variables and categorical weather variables on time indoors away-from-home, and 12-day lagged hospitalizations.**

|  | **Daily county-mean percent of time spent indoors, away from home** | | | **12-day lagged hospitalization admissions (mean-centered by county-season)** | | |
| --- | --- | --- | --- | --- | --- | --- |
| **Control variables** ^a^ | **β** | **95 % CI** | **p-value** | **β** | **95 % CI** | **p-value** |
| **Weekend/holiday (yes/no)** |  |  |  |  |  |  |
| All Seasons ^b^ | -0.98 | -1.04 - -0.91 | <0.001* | -0.22 | -0.56 – 0.12 | 0.201 |
| Spring | -1.22 | -1.40 - -1.04 | <0.001* | 0.06 | -0.51 – 0.63 | 0.835 |
| Summer | -0.97 | -1.04 - -0.91 | <0.001* | 0.28 | -0.03 – 0.60 | 0.080 |
| Fall | -1.18 | -1.28 - -1.09 | <0.001* | -0.24 | -1.09 – 0.62 | 0.584 |
| Winter | -1.04 | -1.17 - -0.91 | <0.001* | -0.67 | -1.49 – 0.15 | 0.109 |
| **Stay-at-home order (yes/no)** |  |  |  |  |  |  |
| All Seasons ^b^ | -0.39 | -0.53 - -0.26 | <0.001* | 0.19 | -0.32 – 0.69 | 0.469 |
| Spring | -0.31 | -0.55 - -0.08 | 0.009* | 0.28 | -0.26 – 0.82 | 0.305 |
| Summer | NA | NA | NA | NA | NA | NA |
| Fall | NA | NA | NA | NA | NA | NA |
| Winter | NA | NA | NA | NA | NA | NA |
| **Rising Colorado hospitalizations (yes/no)** |  |  |  |  |  |  |
| All Seasons ^b^ | -0.11 | -0.19 – -0.03 | 0.005* | 1.15 | 0.81 – 1.49 | <0.001* |
| Spring | -0.30 | -0.49 - -0.10 | 0.004* | 1.17 | 0.58 – 1.76 | <0.001* |
| Summer | 0.07 | -0.05 – 0.18 | 0.253 | 0.27 | -0.08 – 0.62 | 0.131 |
| Fall | -0.01 | -0.14 – 0.13 | 0.904 | 2.50 | 1.55 – 3.45 | <0.001* |
| Winter | -0.31 | -0.50 - -0.12 | 0.001* | 1.58 | 0.61 – 2.55 | 0.001* |
|  |  |  |  |  |  |  |
| **Categorical weather conditions** ^c^ | **β** | **95 % CI** | **p-value** | **β** | **95 % CI** | **p-value** |
| **Minimum temperature** |  |  |  |  |  |  |
| All season ^b^ |  |  |  |  |  |  |
| <-0.5 vs. mid | 0.09 | 0.01 – 0.17 | 0.028* | 0.14 | -0.25 – 0.53 | 0.490 |
| >0.5 vs. mid | -0.09 | -0.16 - -0.01 | 0.020* ^d^ | -0.41 | -0.78 - -0.03 | 0.034* ^d^ |
| Spring |  |  |  |  |  |  |
| <-0.5 vs. mid | 0.30 | 0.11 – 0.48 | 0.002* | -0.18 | -0.78 – 0.42 | 0.557 |
| >0.5 vs. mid | -0.19 | -0.41 – 0.04 | 0.099 | -0.23 | -0.96 – 0.49 | 0.526 |
| Summer |  |  |  |  |  |  |
| <-0.5 vs. mid | -0.08 | -0.16 - -0.00 | 0.039* | -0.35 | -0.77 – 0.07 | 0.102 |
| >0.5 vs. mid | -0.09 | -0.16 - -0.03 | 0.004* | -0.14 | -0.47 – 0.19 | 0.394 |
| Fall |  |  |  |  |  |  |
| <-1 vs. mid | -0.05 | -0.18 – 0.08 | 0.452 | 0.61 | -0.56 – 1.78 | 0.306 |
| >1 vs. mid | -0.08 | -0.20 – 0.04 | 0.210 | -0.03 | -1.12 – 1.06 | 0.959 |
| Winter |  |  |  |  |  |  |
| <-1 vs. mid | -0.32 | -0.47 – -0.17 | <0.001* | 0.26 | -0.69 – 1.21 | 0.589 |
| >1 vs. mid | 0.04 | -0.13 – 0.22 | 0.640 | 1.19 | -0.01 – 2.40 | 0.053 |
| **Maximum temperature** |  |  |  |  |  |  |
| All season ^b^ |  |  |  |  |  |  |
| <-1 vs. mid | 0.08 | -0.00 – 0.17 | 0.060 | -0.19 | -0.62 – 0.23 | 0.374 |
| >1 vs. mid | -0.11 | -0.20 – 0.01 | 0.024* | -0.28 | -0.74 – 0.19 | 0.241 |
| Spring |  |  |  |  |  |  |
| <-1 vs mid | 0.23 | 0.03 – 0.44 | 0.028* ^d^ | -0.73 | -1.41 - -0.04 | 0.037* ^d^ |
| >1 vs mid | -0.12 | -0.42 – 0.18 | 0.448 | -0.62 | -1.58 – 0.33 | 0.201 |
| Summer |  |  |  |  |  |  |
| <-1 vs. mid | -0.05 | -0.14 – 0.04 | 0.303 | -0.48 | -0.94 - -0.02 | 0.039* |
| >1 vs. mid | -0.08 | -0.16 – -0.00 | 0.954 | -0.18 | -0.59 – 0.23 | 0.390 |
| Fall |  |  |  |  |  |  |
| <-1 vs. mid | -0.09 | -0.21 – 0.03 | 0.145 | 0.32 | -0.78 – 1.41 | 0.567 |
| >1 vs. mid | -0.13 | -0.26 - -0.01 | 0.041* ^d^ | -0.28 | -1.42 – 0.86 | 0.632 |
| Winter |  |  |  |  |  |  |
| <-1 vs. mid | -0.23 | -0.37 - -0.08 | 0.003* | 0.40 | -0.59 – 1.39 | 0.431 |
| >1 vs. mid | 0.13 | -0.03 – 0.30 | 0.120 | 0.23 | -0.91 – 1.37 | 0.691 |
| **Minimum Relative Humidity** |  |  |  |  |  |  |
| All season ^b^ |  |  |  |  |  |  |
| <-1 vs. mid | -0.09 | -0.21 – 0.02 | 0.100 | -0.17 | -0.73 – 0.39 | 0.557 |
| >1 vs. mid | -0.06 | -0.15 – 0.03 | 0.209 | -0.27 | -0.72 – 0.18 | 0.238 |
| Spring |  |  |  |  |  |  |
| <-0.5 vs. mid | -0.35 | -0.52 - -0.18 | <0.001* | -0.03 | -0.60 – 0.55 | 0.926 |
| >1 vs. mid | -0.22 | -0.44 - -0.00 | 0.048* | -0.65 | -1.40 – 0.10 | 0.089 |
| Summer |  |  |  |  |  |  |
| <-0.5 vs. mid | 0.02 | -0.04 – 0.08 | 0.435 | 0.12 | -0.19 – 0.44 | 0.439 |
| >1 vs. mid | -0.01 | -0.09 – 0.08 | 0.880 | 0.02 | -0.43 – 0.47 | 0.930 |
| Fall |  |  |  |  |  |  |
| <-0.5 vs. mid | -0.03 | -0.13 – 0.06 | 0.474 | -0.05 | -0.90 – 0.79 | 0.899 |
| >1 vs. mid | -0.19 | -0.33 - -0.05 | 0.008* | 0.39 | -0.86 – 1.64 | 0.540 |
| Winter |  |  |  |  |  |  |
| <-1 vs. mid | 0.23 | 0.04 – 0.43 | 0.018* | -1.04 | -2.36 – 0.27 | 0.121 |
| >1 vs. mid | -0.27 | -0.43 - -0.11 | 0.001* | -0.01 | -1.11 – 1.09 | 0.985 |
| **Maximum relative humidity** |  |  |  |  |  |  |
| All Season ^b^ |  |  |  |  |  |  |
| <-1 vs. mid | -0.11 | -0.19 - -0.02 | 0.015* | -0.42 | -0.85 – 0.12 | 0.057 |
| >1 vs. mid | -0.03 | -0.11 – 0.05 | 0.458 | -0.36 | -0.76 – 0.04 | 0.081 |
| Spring |  |  |  |  |  |  |
| <-1 vs. mid | -0.57 | -0.78 - -0.37 | <0.001* | -0.14 | -0.84 – 0.57 | 0.706 |
| >1 vs. mid | -0.14 | -0.33 – 0.05 | 0.153 | -0.14 | -0.80 – 0.53 | 0.683 |
| Summer |  |  |  |  |  |  |
| <-0.5 vs. mid | 0.06 | -0.00 – 0.12 | 0.072 | 0.01 | -0.31 – 0.33 | 0.963 |
| >1 vs. mid | 0.04 | -0.04 – 0.12 | 0.348 | 0.00 | -0.42 – 0.42 | 0.999 |
| Fall |  |  |  |  |  |  |
| <-0.5 vs. mid | 0.02 | -0.08 – 0.12 | 0.661 | -0.08 | -0.96 – 0.80 | 0.856 |
| >1 vs. mid | -0.03 | -0.15 – 0.08 | 0.570 | -0.02 | -1.07 – 1.04 | 0.974 |
| Winter |  |  |  |  |  |  |
| <-1 vs. mid | 0.23 | 0.05 – 0.41 | 0.014* | -1.00 | -2.23 – 0.24 | 0.113 |
| >1 vs. mid | -0.25 | -0.40 - -0.11 | 0.001* | -0.45 | -1.44 – 0.54 | 0.376 |
| **Minimum absolute humidity** |  |  |  |  |  |  |
| All Season ^b^ |  |  |  |  |  |  |
| <-1 vs. mid | 0.14 | 0.05 – 0.24 | 0.004* | 0.08 | -0.40 – 0.56 | 0.740 |
| >1 vs. mid | -0.09 | -0.18 – 0.00 | 0.053* | -0.31 | -0.77 – 0.15 | 0.183 |
| Spring |  |  |  |  |  |  |
| <-1 vs mid | -0.03 | -0.26 – 0.20 | 0.813 | 0.45 | -0.33 – 1.22 | 0.259 |
| >1 vs mid | -0.38 | -0.63 - -0.13 | 0.003* | 0.07 | -0.76 – 0.90 | 0.865 |
| Summer |  |  |  |  |  |  |
| <-1 vs. mid | 0.07 | -0.02 – 0.15 | 0.133 | -0.03 | -0.48 – 0.41 | 0.892 |
| >1 vs. mid | -0.10 | -0.17 - -0.02 | 0.011* | -0.16 | -0.55 – 0.22 | 0.401 |
| Fall |  |  |  |  |  |  |
| <-1 vs. mid | 0.18 | 0.04 – 0.33 | 0.014* | 0.68 | -0.66 – 2.01 | 0.320 |
| >1 vs. mid | 0.07 | -0.07 – 0.20 | 0.349 | 0.07 | -1.14 – 1.29 | 0.910 |
| Winter |  |  |  |  |  |  |
| <-1 vs. mid | 0.22 | 0.03 – 0.40 | 0.017* ^d^ | -1.28 | -2.50 - -0.07 | 0.039* ^d^ |
| >1 vs. mid | -0.12 | -0.32 – 0.07 | 0.209 | -0.43 | -1.70 – 0.84 | 0.509 |
| **Maximum absolute humidity** |  |  |  |  |  |  |
| All Season ^b^ |  |  |  |  |  |  |
| <-1 vs. mid | -0.72 | -0.87 – -0.57 | <0.001* | 0.07 | -0.37 – 0.50 | 0.763 |
| >1 vs. mid | -0.11 | -0.18 – -0.04 | 0.002* | -0.11 | -0.58 – 0.36 | 0.657 |
| Spring |  |  |  |  |  |  |
| <-1 vs mid | 0.05 | -0.17 – 0.28 | 0.649 | -1.00 | -1.74 – -0.26 | 0.008* |
| >1 vs mid | -0.15 | -0.46 – 0.15 | 0.312 | -0.12 | -1.07 – 0.84 | 0.810 |
| Summer |  |  |  |  |  |  |
| <-1 vs. mid | 0.05 | -0.03 – 0.12 | 0.201 | 0.01 | -0.38 – 0.40 | 0.963 |
| >1 vs. mid | -0.03 | -0.11 – 0.04 | 0.344 | 0.00 | -0.38 – 0.38 | 0.993 |
| Fall |  |  |  |  |  |  |
| <-1 vs. mid | -0.09 | -0.23 – 0.06 | 0.251 | 1.27 | -0.04 – 2.57 | 0.058 |
| >1 vs. mid | -0.03 | -0.16 – 0.09 | 0.611 | 0.23 | -0.92 – 1.37 | 0.698 |
| Winter |  |  |  |  |  |  |
| <-1 vs. mid | -0.13 | -0.28 – 0.03 | 0.106 | 0.05 | -0.99 – 1.08 | 0.931 |
| >1 vs. mid | -0.06 | -0.31 – 0.18 | 0.619 | 0.05 | -1.56 – 1.67 | 0.950 |
| **Wind Speed** |  |  |  |  |  |  |
| All Season ^b^ |  |  |  |  |  |  |
| <-1 vs. mid | -0.01 | -0.11 – 0.08 | 0.806 | -0.09 | -0.57 – 0.40 | 0.730 |
| >1 vs. mid | 0.03 | -0.06 – 0.12 | 0.469 | 0.39 | -0.06 – 0.84 | 0.093 |
| Spring |  |  |  |  |  |  |
| <-1 vs mid | -0.04 | -0.27 – 0.19 | 0.753 | 0.13 | -0.64 – 0.90 | 0.745 |
| >1 vs mid | 0.10 | -0.12 – 0.32 | 0.360 | 0.03 | -0.70 – 0.76 | 0.930 |
| Summer |  |  |  |  |  |  |
| <-1 vs. mid | -0.00 | -0.09 – 0.09 | 0.950 | -0.09 | -0.57 – 0.40 | 0.725 |
| >1 vs. mid | -0.08 | -0.16 – 0.01 | 0.075 | -0.40 | -0.83 – 0.04 | 0.074 |
| Fall |  |  |  |  |  |  |
| <-1 vs. mid | -0.08 | -0.22 – 0.06 | 0.254 | -0.03 | -1.26 – 1.21 | 0.966 |
| >1 vs. mid | -0.16 | -0.29 - -0.03 | 0.018* | 1.11 | -0.08 – 2.29 | 0.067 |
| Winter |  |  |  |  |  |  |
| <-1 vs. mid | 0.15 | -0.02 – 0.33 | 0.092 | -0.25 | -1.41 – 0.93 | 0.683 |
| >1 vs. mid | 0.11 | -0.07 – 0.29 | 0.235 | -0.26 | -1.43 – 0.91 | 0.665 |
| **Precipitation** |  |  |  |  |  |  |
| All Season ^b^ |  |  |  |  |  |  |
| <-0.5 vs. mid | -0.04 | -0.17 – 0.10 | 0.614 | 0.01 | -0.68 – 0.68 | 0.987 |
| >0.5 vs. mid | -0.04 | -0.14 – 0.05 | 0.386 | -0.06 | -0.56 – 0.43 | 0.801 |
| Spring |  |  |  |  |  |  |
| <-0.30 vs mid | -0.10 | -0.27 – 0.06 | 0.223 | -0.13 | -0.68 – 0.42 | 0.646 |
| >0.5 vs mid | -0.24 | -0.50 – 0.03 | 0.084 | 0.03 | -0.87 – 0.93 | 0.945 |
| Summer |  |  |  |  |  |  |
| <-0.5 vs. mid | 0.05 | -0.02 – 0.13 | 0.175 | 0.40 | 0.00 – 0.79 | 0.047* |
| >0.5 vs. mid | -0.04 | -0.12 – 0.04 | 0.371 | 0.40 | -0.02 – 0.81 | 0.061 |
| Fall |  |  |  |  |  |  |
| <-0.5 vs. mid | 0.07 | -0.25 – 0.39 | 0.665 | -0.85 | -3.70 – 2.00 | 0.557 |
| >0.5 vs. mid | 0.07 | -0.09 – 0.23 | 0.369 | -0.22 | -1.65 – 1.21 | 0.763 |
| Winter |  |  |  |  |  |  |
| <-0.5 vs. mid | -0.02 | -0.29 – 0.25 | 0.894 | -0.34 | -2.14 – 1.46 | 0.712 |
| >0.5 vs. mid | -0.24 | -0.43 - -0.05 | 0.012* | -0.15 | -1.42 – 1.11 | 0.811 |
| **Solar Radiation** |  |  |  |  |  |  |
| All Season ^b^ |  |  |  |  |  |  |
| <-1.5 vs. mid | 0.02 | -0.11 – 0.14 | 0.798 | -0.77 | -1.41 - -0.14 | 0.017* |
| >0 vs. mid | -0.12 | -0.19 - -0.05 | 0.001* ^d^ | -0.74 | -1.09 - -0.39 | <0.001* ^d^ |
| Spring |  |  |  |  |  |  |
| <-0.5 vs mid | 0.39 | 0.16 – 0.62 | 0.001* | -0.49 | -1.20 – 0.23 | 0.183 |
| >0.5 vs mid | -0.37 | -0.62 - -0.11 | 0.004* | -0.26 | -1.09 – 0.58 | 0.544 |
| Summer |  |  |  |  |  |  |
| <-1 vs. mid | -0.07 | -0.16 – 0.02 | 0.129 | 0.03 | -0.47 – 0.54 | 0.903 |
| >1 vs. mid | -0.21 | -0.27 - -0.14 | <0.001* | -0.14 | -0.48 – 0.19 | 0.396 |
| Fall |  |  |  |  |  |  |
| <-1.5 vs. mid | 0.03 | -0.16 – 0.21 | 0.793 | -1.24 | -2.92 – 0.43 | 0.146 |
| >0 vs. mid | -0.01 | -0.10 – 0.08 | 0.795 | -1.49 | -2.39 - -0.61 | 0.001* |
| Winter |  |  |  |  |  |  |
| <-1.5 vs. mid | -0.11 | -0.31 – 0.10 | 0.317 | -0.78 | -2.18 – 0.62 | 0.276 |
| >0 vs. mid | 0.26 | 0.13 – 0.39 | <0.001* ^d^ | -1.07 | -1.94 - -0.20 | 0.016* ^d^ |
|  |  |  |  |  |  |  |

β = Beta coefficient

CI = Confident Interval

***** p-value < 0.05

^a^ The beta coefficient, 95% confidence interval and p-value presented for each control variable correspond with the linear regression models assessing the impact of each on the mean percent of time indoors away from home (left) and 12-day lagged COVID-19 hospital admissions (right), including an auto-correlation term indicating yesterday’s response variable value

^b^ Models that were not stratified by season instead included season as a covariate to account for season as a confounder

^c^ The beta coefficient, 95% confidence interval and p-value presented for each independent weather variable correspond with the adjusted models assessing the impact of each treatment variable on the mean percent of time indoors away from home (left) and 12-day lagged COVID-19 hospital admissions (right), controlling for holidays and weekends, the stay-at-home order, increasing Colorado hospitalizations, as well as an auto-correlation term indicating yesterday’s response variable’s value

^d^ Weather variables that were associated with both mean percent of time spent indoors away-from-home and 12-day lagged hospitalizations are highlighted in gray, as these criteria was used to determine which variables to assess in the mediation analysis
