# Supplementary material for: Does behavior mediate the effect of weather on SARS-CoV-2 transmission? evidence from cell-phone data
Source: PLoS One. 2024 Jun 21;19(6):e0305323. doi: 10.1371/journal.pone.0305323 (PMC11192350; doi:10.1371/journal.pone.0305323)
Supplement: S2 Table — (DOCX) [file pone.0305323.s002.docx]

**Table S2. Sensitivity analysis results of the linear regression results using categorical weather conditions on time at home and COVID hospitalizations.**

|  | **Daily county-mean percent of time spent at home (indoors or out)** | | | **12-day lagged hospitalization admissions (mean-centered by county-season)** | | |
| --- | --- | --- | --- | --- | --- | --- |
| **Control variables** ^a^ | **β** | **95% CI** | **p-value** | **β** | **95 % CI** | **p-value** |
| **Minimum temperature** |  |  |  |  |  |  |
| **All season** ^b^ |  |  |  |  |  |  |
| **Control variables** ^a^ | **β** | **95 % CI** | **p-value** | **β** | **95 % CI** | **p-value** |
| **Weekend/holiday (yes/no)** |  |  |  |  |  |  |
| All Seasons ^b^ | 1.01 | 0.89 – 1.14 | <0.001* | -0.22 | -0.56 – 0.12 | 0.201 |
| Spring | 1.46 | 1.20 – 1.73 | <0.001* | 0.06 | -0.51 – 0.63 | 0.835 |
| Summer | 0.34 | 1.15 – 0.53 | <0.001* | 0.28 | -0.03 – 0.60 | 0.080 |
| Fall | 0.60 | 0.36 – 0.85 | <0.001* | -0.24 | -1.09 – 0.62 | 0.584 |
| Winter | 1.44 | 1.17 – 1.71 | <0.001* | -0.67 | -1.49 – 0.15 | 0.109 |
| **Stay-at-home order (yes/no)** |  |  |  |  |  |  |
| All Seasons ^b^ | 1.00 | 0.69 – 1.31 | <0.001* | 0.19 | -0.32 – 0.69 | 0.469 |
| Spring | 0.46 | 0.08 – 0.85 | 0.019* | 0.28 | -0.26 – 0.82 | 0.305 |
| Summer | NA | NA | NA | NA | NA | NA |
| Fall | NA | NA | NA | NA | NA | NA |
| Winter | NA | NA | NA | NA | NA | NA |
| **Rising Colorado hospitalizations (yes/no)** |  |  |  |  |  |  |
| All Seasons ^b^ | 0.27 | 0.13 – 0.41 | <0.001* | 1.15 | 0.81 – 1.49 | <0.001* |
| Spring | 0.54 | 0.25 – 0.84 | <0.001* | 1.17 | 0.58 – 1.76 | <0.001* |
| Summer | -0.06 | -0.25 – 0.14 | 0.566 | 0.27 | -0.08 – 0.62 | 0.131 |
| Fall | 0.19 | -0.05 – 0.43 | 0.118 | 2.50 | 1.55 – 3.45 | <0.001* |
| Winter | 0.82 | 0.45 – 1.19 | <0.001* | 1.58 | 0.61 – 2.55 | 0.001* |
|  |  |  |  |  |  |  |
| **Categorical weather conditions** ^c^ | **β** | **95% CI** | **p-value** | **β** | **95 % CI** | **p-value** |
| **Minimum temperature** |  |  |  |  |  |  |
| **All season** ^b^ |  |  |  |  |  |  |
| <-0.5 vs. mid | 0.09 | -0.05 – 0.23 | 0.225 | 0.14 | -0.25 – 0.53 | 0.490 |
| >0.5 vs. mid | -0.08 | -0.22 – 0.05 | 0.239 | -0.41 | -0.78 - -0.03 | 0.034* |
| Spring |  |  |  |  |  |  |
| <-0.5 vs. mid | -0.04 | -0.32 – 0.24 | 0.782 | -0.18 | -0.78 – 0.42 | 0.557 |
| >0.5 vs. mid | 0.04 | -0.30 – 0.39 | 0.816 | -0.23 | -0.96 – 0.49 | 0.526 |
| Summer |  |  |  |  |  |  |
| <-0.5 vs. mid | 0.13 | -0.11 – 0.38 | 0.285 | -0.35 | -0.77 – 0.07 | 0.102 |
| >0.5 vs. mid | -0.22 | -0.41 - -0.03 | 0.022* | -0.14 | -0.47 – 0.19 | 0.394 |
| Fall |  |  |  |  |  |  |
| <-1 vs. mid | 0.86 | 0.51 – 1.20 | <0.001* | 0.61 | -0.56 – 1.78 | 0.306 |
| >1 vs. mid | -0.15 | -0.47 – 0.16 | 0.342 | -0.03 | -1.12 – 1.06 | 0.959 |
| Winter |  |  |  |  |  |  |
| <-1 vs. mid | 0.87 | 0.56 – 1,18 | <0.001* | 0.26 | -0.69 – 1.21 | 0.589 |
| >1 vs. mid | 0.15 | -0.21 – 0.51 | 0.417 | 1.19 | -0.01 – 2.40 | 0.053 |
| **Maximum temperature** |  |  |  |  |  |  |
| All season ^b^ |  |  |  |  |  |  |
| <-1 vs. mid | 0.52 | 0.36 – 0.67 | <0.001* | -0.19 | -0.62 – 0.23 | 0.374 |
| >1 vs. mid | -0.04 | -0.21 – 0.13 | 0.633 | -0.28 | -0.74 – 0.19 | 0.241 |
| Spring |  |  |  |  |  |  |
| <-1 vs mid | 0.54 | 0.23 – 0.86 | 0.001* ^d^ | -0.73 | -1.41 - -0.04 | 0.037* ^d^ |
| >1 vs mid | 0.19 | -0.27 – 0.64 | 0.421 | -0.62 | -1.58 – 0.33 | 0.201 |
| Summer |  |  |  |  |  |  |
| <-1 vs. mid | 0.23 | -0.03 – 0.48 | 0.080 | -0.48 | -0.94 - -0.02 | 0.039* |
| >1 vs. mid | -0.70 | -0.93 - -0.47 | <0.001* | -0.18 | -0.59 – 0.23 | 0.390 |
| Fall |  |  |  |  |  |  |
| <-1 vs. mid | 1.10 | 0.78 – 1.42 | <0.001* | 0.32 | -0.78 – 1.41 | 0.567 |
| >1 vs. mid | -0.14 | -0.46 – 0.18 | 0.389 | -0.28 | -1.42 – 0.86 | 0.632 |
| Winter |  |  |  |  |  |  |
| <-1 vs. mid | 0.88 | 0.58 – 1.18 | <0.001* | 0.40 | -0.59 – 1.39 | 0.431 |
| >1 vs. mid | -0.08 | -0.43 – 0.27 | 0.653 | 0.23 | -0.91 – 1.37 | 0.691 |
| **Minimum Relative Humidity** |  |  |  |  |  |  |
| All season ^b^ |  |  |  |  |  |  |
| <-1 vs. mid | 0.06 | -0.14 – 0.26 | 0.588 | -0.17 | -0.73 – 0.39 | 0.557 |
| >1 vs. mid | 0.75 | 0.59 – 0.91 | <0.001* | -0.27 | -0.72 – 0.18 | 0.238 |
| Spring |  |  |  |  |  |  |
| <-0.5 vs. mid | 0.13 | -0.13 – 0.38 | 0.328 | -0.03 | -0.60 – 0.55 | 0.926 |
| >1 vs. mid | 1.12 | 0.79 – 1.45 | <0.001* | -0.65 | -1.40 – 0.10 | 0.089 |
| Summer |  |  |  |  |  |  |
| <-0.5 vs. mid | -0.28 | -0.45 - -0.10 | 0.002** | 0.12 | -0.19 – 0.44 | 0.439 |
| >1 vs. mid | 0.20 | -0.05 – 0.45 | 0.118 | 0.02 | -0.43 – 0.47 | 0.930 |
| Fall |  |  |  |  |  |  |
| <-0.5 vs. mid | 0.02 | -0.22 – 0.26 | 0.866 | -0.05 | -0.90 – 0.79 | 0.899 |
| >1 vs. mid | 1.30 | 0.96 – 1.64 | <0.001* | 0.39 | -0.86 – 1.64 | 0.540 |
| Winter |  |  |  |  |  |  |
| <-1 vs. mid | -0.07 | -0.48 – 0.34 | 0.746 | -1.04 | -2.36 – 0.27 | 0.121 |
| >1 vs. mid | 0.79 | 0.44 – 1.13 | <0.001* | -0.01 | -1.11 – 1.09 | 0.985 |
| **Maximum relative humidity** |  |  |  |  |  |  |
| All Season ^b^ |  |  |  |  |  |  |
| <-1 vs. mid | 0.09 | -0.07 – 0.24 | 0.274 | -0.42 | -0.85 – 0.12 | 0.057 |
| >1 vs. mid | 0.44 | 0.30 – 0.59 | <0.001* | -0.36 | -0.76 – 0.04 | 0.081 |
| Spring |  |  |  |  |  |  |
| <-1 vs. mid | 0.42 | 0.10 – 0.74 | 0.009* | -0.14 | -0.84 – 0.57 | 0.706 |
| >1 vs. mid | 0.73 | 0.44 – 1.03 | <0.001* | -0.14 | -0.80 – 0.53 | 0.683 |
| Summer |  |  |  |  |  |  |
| <-0.5 vs. mid | -0.28 | -0.46 - -0.10 | 0.002* | 0.01 | -0.31 – 0.33 | 0.963 |
| >1 vs. mid | 0.01 | -0.23 – 0.24 | 0.958 | 0.00 | -0.42 – 0.42 | 0.999 |
| Fall |  |  |  |  |  |  |
| <-0.5 vs. mid | -0.06 | -0.32 – 0.19 | 0.638 | -0.08 | -0.96 – 0.80 | 0.856 |
| >1 vs. mid | 0.57 | 0.27 – 0.88 | <0.001* | -0.02 | -1.07 – 1.04 | 0.974 |
| Winter |  |  |  |  |  |  |
| <-1 vs. mid | 0.06 | -0.32 – 0.44 | 0.764 | -1.00 | -2.23 – 0.24 | 0.113 |
| >1 vs. mid | 0.73 | 0.42 – 1.04 | <0.001* | -0.45 | -1.44 – 0.54 | 0.376 |
| **Minimum absolute humidity** |  |  |  |  |  |  |
| All Season ^b^ |  |  |  |  |  |  |
| <-1 vs. mid | -0.23 | -0.40 – -0.06 | 0.010* | 0.08 | -0.40 – 0.56 | 0.740 |
| >1 vs. mid | 0.37 | 0.20 – 0.54 | <0.001* | -0.31 | -0.77 – 0.15 | 0.183 |
| Spring |  |  |  |  |  |  |
| <-1 vs mid | -0.28 | -0.63 – 0.07 | 0.117 | 0.45 | -0.33 – 1.22 | 0.259 |
| >1 vs mid | 1.01 | 0.64 – 1.38 | <0.001* | 0.07 | -0.76 – 0.90 | 0.865 |
| Summer |  |  |  |  |  |  |
| <-1 vs. mid | 0.05 | -0.20 – 0.30 | 0.681 | -0.03 | -0.48 – 0.41 | 0.892 |
| >1 vs. mid | 0.18 | -0.05 – 0.40 | 0.120 | -0.16 | -0.55 – 0.22 | 0.401 |
| Fall |  |  |  |  |  |  |
| <-1 vs. mid | -0.26 | -0.64 – 0.12 | 0.186 | 0.68 | -0.66 – 2.01 | 0.320 |
| >1 vs. mid | 0.18 | -0.17 – 0.54 | 0.309 | 0.07 | -1.14 – 1.29 | 0.910 |
| Winter |  |  |  |  |  |  |
| <-1 vs. mid | -0.35 | -0.72 – 0.03 | 0.069 | -1.28 | -2.50 - -0.07 | 0.039* |
| >1 vs. mid | 0.40 | -0.00 – 0.80 | 0.051 | -0.43 | -1.70 – 0.84 | 0.509 |
| **Maximum absolute humidity** |  |  |  |  |  |  |
| All Season ^b^ |  |  |  |  |  |  |
| <-1 vs. mid | 0.31 | 0.15 – 0.47 | <0.001* | 0.07 | -0.37 – 0.50 | 0.763 |
| >1 vs. mid | -0.08 | -0.25 – 0.09 | 0.364 | -0.11 | -0.58 – 0.36 | 0.657 |
| Spring |  |  |  |  |  |  |
| <-1 vs mid | 0.55 | 0.21 – 0.89 | 0.002* ^d^ | -1.00 | -1.74 – -0.26 | 0.008* ^d^ |
| >1 vs mid | 0.33 | -0.12 – 0.78 | 0.147 | -0.12 | -1.07 – 0.84 | 0.810 |
| Summer |  |  |  |  |  |  |
| <-1 vs. mid | -0.13 | -0.35 – 0.09 | 0.233 | 0.01 | -0.38 – 0.40 | 0.963 |
| >1 vs. mid | -0.37 | -0.58 – -0.15 | 0.001* | 0.00 | -0.38 – 0.38 | 0.993 |
| Fall |  |  |  |  |  |  |
| <-1 vs. mid | 1.41 | 1.03 – 1.78 | <0.001* | 1.27 | -0.04 – 2.57 | 0.058 |
| >1 vs. mid | -0.21 | -0.52 – 0.11 | 0.197 | 0.23 | -0.92 – 1.37 | 0.698 |
| Winter |  |  |  |  |  |  |
| <-1 vs. mid | 0.43 | 0.11 – 0.75 | 0.008* | 0.05 | -0.99 – 1.08 | 0.931 |
| >1 vs. mid | -0.50 | -1.00 – 0.00 | 0.050* | 0.05 | -1.56 – 1.67 | 0.950 |
| **Wind Speed** |  |  |  |  |  |  |
| All Season ^b^ |  |  |  |  |  |  |
| <-1 vs. mid | 0.15 | -0.03 – 0.32 | 0.102 | -0.09 | -0.57 – 0.40 | 0.730 |
| >1 vs. mid | 0.07 | -0.09 – 0.24 | 0.376 | 0.39 | -0.06 – 0.84 | 0.093 |
| Spring |  |  |  |  |  |  |
| <-1 vs mid | -0.01 | -0.37 – 0.35 | 0.949 | 0.13 | -0.64 – 0.90 | 0.745 |
| >1 vs mid | -0.06 | -0.40 – 0.28 | 0.746 | 0.03 | -0.70 – 0.76 | 0.930 |
| Summer |  |  |  |  |  |  |
| <-1 vs. mid | -0.21 | -0.47 – 0.06 | 0.133 | -0.09 | -0.57 – 0.40 | 0.725 |
| >1 vs. mid | 0.38 | 0.13 – 0.64 | 0.003* | -0.40 | -0.83 – 0.04 | 0.074 |
| Fall |  |  |  |  |  |  |
| <-1 vs. mid | 0.61 | 0.26 – 0.96 | 0.001* | -0.03 | -1.26 – 1.21 | 0.966 |
| >1 vs. mid | 0.64 | 0.30 – 0.97 | <0.001* | 1.11 | -0.08 – 2.29 | 0.067 |
| Winter |  |  |  |  |  |  |
| <-1 vs. mid | -0.01 | -0.38 – 0.36 | 0.968 | -0.25 | -1.41 – 0.93 | 0.683 |
| >1 vs. mid | -0.41 | -0.78 - -0.03 | 0.034* | -0.26 | -1.43 – 0.91 | 0.665 |
| **Precipitation** |  |  |  |  |  |  |
| All Season ^b^ |  |  |  |  |  |  |
| <-0.5 vs. mid | 0.09 | -0.16 – 0.33 | 0.487 | 0.01 | -0.68 – 0.68 | 0.987 |
| >0.5 vs. mid | 0.50 | 0.32 – 0.68 | <0.001* | -0.06 | -0.56 – 0.43 | 0.801 |
| Spring |  |  |  |  |  |  |
| <-0.30 vs mid | -0.10 | -0.35 – 0.15 | 0.436 | -0.13 | -0.68 – 0.42 | 0.646 |
| >0.5 vs mid | 0.10 | 0.57 – 1.40 | <0.001 | 0.03 | -0.87 – 0.93 | 0.945 |
| Summer |  |  |  |  |  |  |
| <-0.5 vs. mid | -0.01 | -0.24 – 0.21 | 0.908 | 0.40 | 0.00 – 0.79 | 0.047* |
| >0.5 vs. mid | 0.16 | -0.07 – 0.40 | 0.172 | 0.40 | -0.02 – 0.81 | 0.061 |
| Fall |  |  |  |  |  |  |
| <-0.5 vs. mid | 0.34 | -0.47 – 1.15 | 0.406 | -0.85 | -3.70 – 2.00 | 0.557 |
| >0.5 vs. mid | 0.80 | 0.39 – 1.21 | <0.001* | -0.22 | -1.65 – 1.21 | 0.763 |
| Winter |  |  |  |  |  |  |
| <-0.5 vs. mid | 0.14 | -0.42 – 0.70 | 0.619 | -0.34 | -2.14 – 1.46 | 0.712 |
| >0.5 vs. mid | 0.68 | 0.28 – 1.08 | 0.001* | -0.15 | -1.42 – 1.11 | 0.811 |
| **Solar Radiation** |  |  |  |  |  |  |
| All Season ^b^ |  |  |  |  |  |  |
| <-1.5 vs. mid | 0.38 | 0.15 – 0.61 | 0.001* ^d^ | -0.77 | -1.41 - -0.14 | 0.017* ^d^ |
| >0 vs. mid | -0.16 | -0.28 - -0.04 | 0.012* ^d^ | -0.74 | -1.09 - -0.39 | <0.001* ^d^ |
| Spring |  |  |  |  |  |  |
| <-0.5 vs mid | 0.25 | -0.12 – 0.62 | 0.183 | -0.49 | -1.20 – 0.23 | 0.183 |
| >0.5 vs mid | 0.35 | -0.04 – 0.74 | 0.077 | -0.26 | -1.09 – 0.58 | 0.544 |
| Summer |  |  |  |  |  |  |
| <-1 vs. mid | 0.17 | -0.11 – 0.45 | 0.240 | 0.03 | -0.47 – 0.54 | 0.903 |
| >1 vs. mid | -0.29 | -0.47 - -0.10 | 0.002* | -0.14 | -0.48 – 0.19 | 0.396 |
| Fall |  |  |  |  |  |  |
| <-1.5 vs. mid | 0.51 | 0.05 – 0.98 | 0.030* | -1.24 | -2.92 – 0.43 | 0.146 |
| >0 vs. mid | -0.81 | -1.07 - -0.55 | <0.001* ^d^ | -1.49 | -2.39 - -0.61 | 0.001* ^d^ |
| Winter |  |  |  |  |  |  |
| <-1.5 vs. mid | 0.37 | -0.07 – 0.81 | 0.097 | -0.78 | -2.18 – 0.62 | 0.276 |
| >0 vs. mid | -0.43 | -0.70 - -0.15 | 0.002* ^d^ | -1.07 | -1.94 - -0.20 | 0.016* ^d^ |

β = Beta coefficient

CI = Confident Interval

***** p-value < 0.05

^a^ The beta coefficient, 95% confidence interval and p-value presented for each control variable correspond with the linear regression models assessing the impact of each on the mean percent of time at home (left) and 12-day lagged COVID-19 hospital admissions (right), including an auto-correlation term indicating yesterday’s response variable value

^b^ Models that were not stratified by season instead included season as a covariate to account for season as a confounder

^c^ The beta coefficient, 95% confidence interval and p-value presented for each independent weather variable correspond with the adjusted models assessing the impact of each treatment variable on the mean percent of time at home (left) and 12-day lagged COVID-19 hospital admissions (right), controlling for holidays and weekends, the stay-at-home order, increasing Colorado hospitalizations, as well as an auto-correlation term indicating yesterday’s response variable’s value

^d^ Weather variables that were associated with both mean percent of time at home and 12-day lagged hospitalizations are highlighted in gray, as these criteria was used to determine which variables to assess in the mediation analysis
