# Supplementary material for: Does behavior mediate the effect of weather on SARS-CoV-2 transmission? evidence from cell-phone data
Source: PLoS One. 2024 Jun 21;19(6):e0305323. doi: 10.1371/journal.pone.0305323 (PMC11192350; doi:10.1371/journal.pone.0305323)
Supplement: S3 Table — (DOCX) [file pone.0305323.s003.docx]

**Table S3. Sensitivity analysis detailing mediation results of categorical weather conditions and time at home as the mediator.**

|  |  |  | **Estimate of the mediating effects of time at home on 12-day lagged COVID-19 hospital admissions** | | |
| --- | --- | --- | --- | --- | --- |
|  |  |  |  | | |
|  | **Treatment level** ^a^ | **Effect** | **β** | **95% CI** | **P-Value** |
| **All Seasons** |  |  |  |  |  |
| High solar radiation | >0 SD vs. -1.5 – 0 SD | Natural Indirect Effect | -0.02 | -0.05 – 0.00 | 0.090 |
|  | >0 SD vs. -1.5 – 0 SD | Natural Direct Effect | -0.76 | -1.08 - -0.43 | <0.001* |
|  | >0 SD vs. -1.5 – 0 SD | Total Effect | -0.78 | -1.10 - -0.45 | <0.001* |
|  |  |  |  |  |  |
| Low solar radiation | <0 SD vs. -1.5 – 0 SD | Natural Indirect Effect | 0.02 | -0.03 – 0.06 | 0.481 |
|  | <0 SD vs. -1.5 – 0 SD | Natural Direct Effect | -0.80 | -1.33 - -0.28 | 0.003* |
|  | <0 SD vs. -1.5 – 0 SD | Total Effect | -0.79 | -1.31 - -0.26 | 0.003* |
| **Spring** |  |  |  |  |  |
| Low maximum temperature | <-1 SD vs. -1 – 1 SD | Natural Indirect Effect | -0.01 | -0.06 – 0.04 | 0.604 |
|  | <-1 SD vs. -1 – 1 SD | Natural Direct Effect | -0.73 | -1.33 - -0.13 | 0.016* |
|  | <-1 SD vs. -1 – 1 SD | Total Effect | -0.74 | -1.35 - -0.14 | 0.016* |
|  |  |  |  |  |  |
| Low maximum absolute humidity | <-1 SD vs. -1 – 1 SD | Natural Indirect Effect | 0.00 | -0.13 – 0.06 | 0.883 |
|  | <-1 SD vs. -1 – 1 SD | Natural Direct Effect | -1.00 | -1.66 – -0.34 | 0.003* |
|  | <-1 SD vs. -1 – 1 SD | Total Effect | -1.00 | -1.67 - -0.32 | 0.004* |
| **Fall** |  |  |  |  |  |
| High solar radiation | >0 SD vs. -1.5 – 0 SD | Natural Indirect Effect | -0.23 | -0.47 – 0.02 | 0.069 |
|  | >0 SD vs. -1.5 – 0 SD | Natural Direct Effect | -0.97 | -1.88 - -0.06 | 0.036* |
|  | >0 SD vs. -1.5 – 0 SD | Total Effect | -1.20 | -2.00 - -0.39 | 0.004* |
| **Winter** |  |  |  |  |  |
| High solar radiation | >0 SD vs. -1.5 – 0 SD | Natural Indirect Effect | -0.14 | -0.30 – 0.02 | 0.084 |
|  | >0 SD vs. -1.5 – 0 SD | Natural Direct Effect | -0.76 | -1.65 – 0.12 | 0.092 |
|  | >0 SD vs. -1.5 – 0 SD | Total Effect | -0.90 | -1.75 - -0.05 | 0.038* |

β = Beta coefficient

CI = Confident Interval

***** p-value < 0.05

^a^ Seasonal weather conditions were categorized into three groups by examining Lowess plots between the weather variable and both the mediator (time at home) and outcome (12-day lagged hospital admissions) in this analysis. Linear regression analyses compared the association of “high” and “low” weather categories (versus the mid-range) on both the mediator and outcome. Those seasonal weather conditions were significantly associated with both are included in this table
