# Supplementary material for: Does behavior mediate the effect of weather on SARS-CoV-2 transmission? evidence from cell-phone data
Source: PLoS One. 2024 Jun 21;19(6):e0305323. doi: 10.1371/journal.pone.0305323 (PMC11192350; doi:10.1371/journal.pone.0305323)
Supplement: S5 Table — (DOCX) [file pone.0305323.s005.docx]

**Table S5. Sensitivity analysis results for the mediation models using continuous weather variables and time indoors away-from-home as the mediator.**

|  |  |  | **Estimating the mediating effects of time spent indoors away-from-home on 12-day lagged COVID hospital admissions** | | |
| --- | --- | --- | --- | --- | --- |
|  |  |  |  | | |
|  | **Treatment level** ^a^ | **Effect** | **β** | **95% CI** | **P-Value** |
| **All Seasons** |  |  |  |  |  |
| Low minimum temperature | -1 SD vs. mean | Natural Indirect Effect | -0.01 | -0.01 – 0.00 | 0.103 |
|  | -1 SD vs. mean | Natural Direct Effect | 0.20 | 0.05 – 0.37 | 0.013* |
|  | -1 SD vs. mean | Total Effect | 0.20 | 0.04 – 0.36 | 0.015* |
|  |  |  |  |  |  |
| High minimum temperature | +1 SD vs. mean | Natural Indirect Effect | 0.01 | -0.00 – 0.02 | 0.137 |
|  | +1 SD vs. mean | Natural Direct Effect | -0.20 | -0.37 – -0.04 | 0.013* |
|  | +1 SD vs. mean | Total Effect | -0.20 | -0.36 – -0.03 | 0.017* |
|  |  |  |  |  |  |

β = Beta coefficient

CI = Confident Interval

***** p-value < 0.05

^a^ Seasonal weather conditions were included in models as continuous standardized measures within the mediation models. The treatment group was defined as ±1 SD, while the control group was defined as the mean.
