# Supplementary material for: Does behavior mediate the effect of weather on SARS-CoV-2 transmission? evidence from cell-phone data
Source: PLoS One. 2024 Jun 21;19(6):e0305323. doi: 10.1371/journal.pone.0305323 (PMC11192350; doi:10.1371/journal.pone.0305323)
Supplement: S6 Table — (DOCX) [file pone.0305323.s006.docx]

**Table S6. Sensitivity analysis detailing linear regression results of continuous weather conditions on time at home, and on hospitalizations.**

|  | **Percent of time at home**  **(mean-centered by county season)** | | | **12-day lagged hospitalization admissions (mean-centered by county-season)** | | |
| --- | --- | --- | --- | --- | --- | --- |
| **Continuous weather conditions** ^a^ | **β** | **95 % CI** | **p-value** | **β** | **95 % CI** | **p-value** |
| **Minimum temperature** |  |  |  |  |  |  |
| **All Seasons** ^b^ | **-0.09** | **-0.15 - -0.03** | **0.004*** ^c^ | **-0.18** | **-0.35 – -0.02** | **0.029*** ^c^ |
| Spring | 0.03 | -0.11 – 0.17 | 0.674 | 0.07 | -0.23 – 0.37 | 0.643 |
| Summer | -0.22 | -0.31 – -0.12 | <0.001* | 0.02 | -0.13 – 0.17 | 0.773 |
| Fall | -0.43 | -0.56 – -0.29 | <0.001* | -0.36 | -0.78 – 0.07 | 0.102 |
| Winter | -0.30 | -0.43 - -0.16 | <0.001* | -0.04 | -0.47 – 0.38 | 0.836 |
| **Maximum temperature** |  |  |  |  |  |  |
| **All Seasons** ^b^ | **-0.17** | **-0.23 – -0.12** | **<0.001*** | **-0.09** | **-0.25 – 0.07** | **0.264** |
| Spring | -0.15 | -0.29 – -0.02 | 0.027* | 0.20 | -0.09 – 0.48 | 0.177 |
| Summer | -0.23 | -0.31 - -0.14 | <0.001* | 0.05 | -0.09 – 0.19 | 0.505 |
| Fall | -0.50 | -0.63 – -0.38 | <0.001* | -0.34 | -0.76 – 0.08 | 0.111 |
| Winter | -0.31 | -0.44 – -0.18 | <0.001* | -0.19 | -0.60 – 0.22 | 0.359 |
| **Minimum relative humidity** |  |  |  |  |  |  |
| **All Seasons** ^b^ | **0.27** | **0.21 – 0.32** | **<0.001*** | **-0.07** | **-0.23 – 0.09** | **0.375** |
| Spring | 0.37 | 0.25 – 0.48 | <0.001* | -0.19 | -0.45 – 0.07 | 0.147 |
| Summer | 0.18 | 0.09 – 0.27 | <0.001* | -0.03 | -0.18 – 0.13 | 0.729 |
| Fall | 0.39 | 0.28 – 0.51 | <0.001* | 0.14 | -0.25 – 0.53 | 0.472 |
| Winter | 0.33 | 0.20 – 0.46 | <0.001* | 0.13 | -0.28 – 0.55 | 0.527 |
| **Maximum relative humidity** |  |  |  |  |  |  |
| **All Seasons** ^b^ | **0.15** | **0.09 – 0.20** | **<0.001*** | **-0.02** | **-0.18 – 0.13** | **0.765** |
| Spring | 0.17 | 0.05 – 0.29 | 0.005* | -0.02 | -0.28 – 0.24 | 0.875 |
| Summer | 0.13 | 0.04 – 0.21 | 0.003* | 0.01 | -0.14 – 0.16 | 0.917 |
| Fall | 0.24 | 0.12 – 0.36 | <0.001* | 0.05 | -0.35 – 0.44 | 0.814 |
| Winter | 0.22 | 0.10 – 0.35 | 0.001* | 0.12 | -0.28 – 0.52 | 0.551 |
| **Minimum absolute humidity** |  |  |  |  |  |  |
| **All Seasons** ^b^ | **0.17** | **0.11 – 0.22** | **<0.001*** | **-0.14** | **-0.30 – 0.01** | **0.075** |
| Spring | 0.40 | 0.27 – 0.53 | <0.001* | -0.16 | -0.45 – 0.13 | 0.269 |
| Summer | 0.07 | -0.01 – 0.16 | 0.095 | -0.05 | -0.19 – 0.09 | 0.498 |
| Fall | 0.11 | -0.01 – 0.23 | 0.062 | 0.05 | -0.36 – 0.45 | 0.826 |
| Winter | 0.17 | 0.03 – 0.31 | 0.017* | 0.11 | -0.33 – 0.56 | 0.620 |
| **Maximum absolute humidity** |  |  |  |  |  |  |
| **All Seasons** ^b^ | **-0.12** | **-0.18 - -0.06** | **<0.001*** | **-0.15** | **-0.31 – 0.02** | **0.084** |
| Spring | -0.07 | -0.23 – 0.09 | 0.390 | 0.21 | -0.11 – 0.53 | 0.201 |
| Summer | -0.13 | -0.21 – -0.05 | 0.001* | 0.03 | -0.10 – 0.17 | 0.646 |
| Fall | -0.46 | -0.60 – -0.32 | <0.001* | -0.38 | -0.85 – 0.10 | 0.121 |
| Winter | -0.24 | -0.38 – -0.11 | 0.001* | -0.14 | -0.58 – 0.30 | 0.545 |
| **Mean shortwave radiation** |  |  |  |  |  |  |
| **All Seasons** ^b^ | **-0.14** | **-0.20 – -0.08** | **<0.001*** | **-0.17** | **-0.34 - -0.00** | **0.056** |
| Spring | -0.09 | -0.25 – 0.07 | 0.264 | 0.08 | -0.22 – 0.39 | 0.581 |
| Summer | -0.11 | -0.21 – -0.02 | 0.017* | -0.05 | -0.22 – 0.12 | 0.547 |
| Fall | -0.51 | -0.63 – -0.39 | <0.001* | -0.33 | -0.73 – 0.07 | 0.106 |
| Winter | -0.31 | -0.45 – -0.16 | <0.001* | -0.43 | -0.89 – 0.03 | 0.065 |
| **Total precipitation** |  |  |  |  |  |  |
| **All Seasons** ^b^ | 0.19 | **0.13 – 0.24** | **<0.001*** | **0.03** | **-0.13 – 0.19** | **0.710** |
| Spring | 0.40 | 0.26 – 0.53 | <0.001* | 0.13 | -0.17 – 0.43 | 0.393 |
| Summer | 0.06 | -0.01 – 0.13 | 0.119 | 0.10 | -0.03 – 0.23 | 0.122 |
| Fall | 0.27 | 0.16 – 0.39 | <0.001* | 0.05 | -0.35 – 0.46 | 0.788 |
| Winter | 0.22 | 0.08 – 0.35 | 0.001* | -0.07 | -0.49 – 0.36 | 0.758 |
| **Mean wind velocity** |  |  |  |  |  |  |
| **All Seasons** ^b^ | **0.00** | **-0.05 – 0.06** | **0.903** | **0.09** | **-0.06 – 0.25** | **0.253** |
| Spring | -0.04 | -0.17 – 0.08 | 0.502 | -0.10 | -0.36 – 0.17 | 0.480 |
| Summer | 0.16 | 0.08 – 0.25 | <0.001* | -0.03 | -0.18 – 0.11 | 0.653 |
| Fall | 0.14 | 0.02 – 0.27 | 0.021* | 0.05 | -0.38 – 0.48 | 0.817 |
| Winter | -0.08 | -0.22 – 0.05 | 0.224 | -0.03 | -0.45 – 0.39 | 0.879 |

β = Beta coefficient

CI = Confident Interval

***** p-value < 0.05

^a^ The beta coefficient, 95% confidence interval and p-value presented for each continuous independent weather variable correspond with the adjusted models assessing the impact of each treatment variable on the mean percent of time indoors at home (left) and 12-day lagged COVID-19 hospital admissions (right), controlling for holidays and weekends, the stay-at-home order, increasing Colorado hospitalizations, as well as an auto-correlation term indicating yesterday’s response variable’s value

^b^ Models that were not stratified by season instead included season as a covariate to account for season as a confounder

^c^ Weather variables that were associated with both mean percent of time at home and 12-day lagged hospitalizations are highlighted in gray, as these criteria was used to determine which variables to assess in the mediation analysis
