# Supplementary material for: Does behavior mediate the effect of weather on SARS-CoV-2 transmission? evidence from cell-phone data
Source: PLoS One. 2024 Jun 21;19(6):e0305323. doi: 10.1371/journal.pone.0305323 (PMC11192350; doi:10.1371/journal.pone.0305323)
Supplement: S7 Table — (DOCX) [file pone.0305323.s007.docx]

**Table S7. Sensitivity analysis detailing mediation results assessing time at home as a mediator between continuous weather conditions and COVID hospital admissions.**

|  |  |  | **Estimating the mediating effects of time spent at home on 12-day lagged COVID hospital admissions** | | |
| --- | --- | --- | --- | --- | --- |
|  |  |  |  | | |
|  | **Treatment level** ^a^ | **Effect** | **β** | **95% CI** | **P-Value** |
| **All Seasons** |  |  |  |  |  |
| Low minimum temperature | -1 SD vs. mean | Natural Indirect Effect | 0.01 | -0.00 – 0.01 | 0.113 |
|  | -1 SD vs. mean | Natural Direct Effect | 0.16 | 0.01 – 0.32 | 0.036* |
|  | -1 SD vs. mean | Total Effect | 0.17 | 0.02 – 0.32 | 0.031* |
|  |  |  |  |  |  |
| High minimum temperature | +1 SD vs. mean | Natural Indirect Effect | -0.01 | -0.02 – 0.00 | 0.103 |
|  | +1 SD vs. mean | Natural Direct Effect | -0.16 | -0.32 – -0.01 | 0.036* |
|  | +1 SD vs. mean | Total Effect | -0.17 | -0.33 – -0.02 | 0.027* |
|  |  |  |  |  |  |

β = Beta coefficient

CI = Confident Interval

***** p-value < 0.05

^a^ Seasonal weather conditions were included in models as continuous standardized measures within the mediation models. The treatment group was defined as ±1 SD, while the control group was defined as the mean.
