# Supplementary material for: Does behavior mediate the effect of weather on SARS-CoV-2 transmission? evidence from cell-phone data
Source: PLoS One. 2024 Jun 21;19(6):e0305323. doi: 10.1371/journal.pone.0305323 (PMC11192350; doi:10.1371/journal.pone.0305323)
Supplement: S8 Table — (DOCX) [file pone.0305323.s008.docx]

**Table S8. Sensitivity analysis detailing mediation results without hospitalization growth, using categorical weather conditions and time indoors away from home as the mediator.**

|  |  |  | **With Hosp Growth** | | | **Without Hosp Growth** | | |
| --- | --- | --- | --- | --- | --- | --- | --- | --- |
|  |  |  |  | | |  | | |
|  | **Treatment level** ^a^ | **Effect** | **β** | **95% CI** | **p-value** | **β** | **95% CI** | **p-value** |
| **All-seasons** |  |  |  |  |  |  |  |  |
|  |  |  |  |  |  |  |  |  |
| High minimum temperature | >0.5 SD vs. -0.5 – 0.5 SD | Natural Indirect Effect | 0.02 | -0.01 – 0.04 | 0.144 | 0.01 | -0.01 – 0.03 | 0.324 |
|  | >0.5 SD vs. -0.5 – 0.5 SD | Natural Direct Effect | -0.46 | -0.82 – -0.11 | 0.011* | -0.54 | -0.90 - -0.18 | 0.003* |
|  | >0.5 SD vs. -0.5 – 0.5 SD | Total Effect | -0.45 | -0.80 – -0.10 | 0.013* | -0.53 | -0.88 - -0.18 | 0.003* |
|  |  |  |  |  |  |  |  |  |
| High solar radiation | >0 SD vs. -1.5 – 0 SD | Natural Indirect Effect | 0.03 | -0.00 – 0.07 | 0.054 | 0.02 | -0.01 – 0.04 | 0.184 |
|  | >0 SD vs. -1.5 – 0 SD | Natural Direct Effect | -0.88 | -1.23 - -0.53 | <0.001* | -1.00 | -1.35 - -0.64 | <0.001* |
|  | >0 SD vs. -1.5 – 0 SD | Total Effect | -0.85 | -1.19 - -0.51 | <0.001* | -0.98 | -1.33 - -0.63 | <0.001* |
| **Winter** |  |  |  |  |  |  |  |  |
|  |  |  |  |  |  |  |  |  |
| Low minimum absolute humidity | <-1 SD vs. -1 – 1 SD | Natural Indirect Effect | -0.10 | -0.35 – 0.16 | 0.462 | -0.11 | -0.29 – 0.07 | 0.248 |
|  | <-1 SD vs. -1 – 1 SD | Natural Direct Effect | -1.19 | -2.44 – 0.06 | 0.063 | -0.60 | -1.67 – 0.47 | 0.274 |
|  | <-1 SD vs. -1 – 1 SD | Total Effect | -1.29 | -2.59 – 0.01 | 0.052 | -0.71 | -1.84 – 0.43 | 0.223 |
|  |  |  |  |  |  |  |  |  |
| High solar radiation | >0 SD vs. -1.5 – 0 SD | Natural Indirect Effect | -0.09 | -0.26 – 0.08 | 0.300 | -0.16 | -0.35 – 0.03 | 0.092 |
|  | >0 SD vs. -1.5 – 0 SD | Natural Direct Effect | -0.95 | -1.82 - -0.09 | 0.030* | -0.91 | -1.69 – 0.01 | 0.054 |
|  | >0 SD vs. -1.5 – 0 SD | Total Effect | -1.04 | -1.87 - -0.21 | 0.014* | -0.87 | -1.83 - -0.17 | 0.019* |
| **Spring** |  |  |  |  |  |  |  |  |
|  |  |  |  |  |  |  |  |  |
| Low maximum temperature | <-1 SD vs. -1 – 1 SD | Natural Indirect Effect | 0.01 | -0.03 – 0.05 | 0.542 | -0.00 | -0.04 – 0.03 | 0.832 |
|  | <-1 SD vs. -1 – 1 SD | Natural Direct Effect | -0.78 | -1.42 - -0.13 | 0.018* | -0.77 | -1.43 - -0.11 | 0.022* |
|  | <-1 SD vs. -1 – 1 SD | Total Effect | -0.76 | -1.39 - -0.14 | 0.017* | -0.77 | -1.41 - -0.13 | 0.019* |

β = Beta coefficient

CI = Confident Interval

***** p-value < 0.05

^a^ Seasonal weather conditions were categorized into three groups by examining Lowess plots between the weather variable and both the mediator (time at home) and outcome (12-day lagged hospital admissions) in this analysis. Linear regression analyses compared the association of “high” and “low” weather categories (versus the mid-range) on both the mediator and outcome. Those seasonal weather conditions were significantly associated with both are included in this table.
