# Supplementary material for: Does behavior mediate the effect of weather on SARS-CoV-2 transmission? evidence from cell-phone data
Source: PLoS One. 2024 Jun 21;19(6):e0305323. doi: 10.1371/journal.pone.0305323 (PMC11192350; doi:10.1371/journal.pone.0305323)
Supplement: S9 Table — (DOCX) [file pone.0305323.s009.docx]

**Table S9. Sensitivity analysis detailing mediation results without hospitalization growth, using categorical weather conditions and time at home as the mediator.**

|  |  |  |  | | |  | | |
| --- | --- | --- | --- | --- | --- | --- | --- | --- |
|  |  |  | **With Hosp Growth** | | | **Without Hosp Growth** | | |
|  | **Treatment level** ^a^ | **Effect** | **β** | **95% CI** | **P-Value** | **β** | **95% CI** | **p-value** |
| **All Seasons** |  |  |  |  |  |  |  |  |
| High solar radiation | >0 SD vs. -1.5 – 0 SD | Natural Indirect Effect | -0.02 | -0.05 – 0.00 | 0.090 | -0.03 | -0.06 – 0.00 | 0.066 |
|  | >0 SD vs. -1.5 – 0 SD | Natural Direct Effect | -0.76 | -1.08 - -0.43 | <0.001* | -0.90 | -1.24 - -0.57 | <0.001* |
|  | >0 SD vs. -1.5 – 0 SD | Total Effect | -0.78 | -1.10 - -0.45 | <0.001* | -0.93 | -1.27 - -0.59 | <0.001* |
|  |  |  |  |  |  |  |  |  |
| Low solar radiation | <0 SD vs. -1.5 – 0 SD | Natural Indirect Effect | 0.02 | -0.03 – 0.06 | 0.481 | 0.02 | -0.02 – 0.06 | 0.362 |
|  | <0 SD vs. -1.5 – 0 SD | Natural Direct Effect | -0.80 | -1.33 - -0.28 | 0.003* | -0.86 | -1.40 - -0.32 | 0.002* |
|  | <0 SD vs. -1.5 – 0 SD | Total Effect | -0.79 | -1.31 - -0.26 | 0.003* | -0.93 | -1.27 - -0.59 | <0.001* |
| **Winter** |  |  |  |  |  |  |  |  |
| High solar radiation | >0 SD vs. -1.5 – 0 SD | Natural Indirect Effect | -0.14 | -0.30 – 0.02 | 0.084 | -0.17 | -0.33 - -0.01 | 0.041* |
|  | >0 SD vs. -1.5 – 0 SD | Natural Direct Effect | -0.76 | -1.65 – 0.12 | 0.092 | -0.65 | -1.51 – 0.22 | 0.143 |
|  | >0 SD vs. -1.5 – 0 SD | Total Effect | -0.90 | -1.75 - -0.05 | 0.038* | -0.81 | -1.66 – 0.03 | 0.060 |
| **Spring** |  |  |  |  |  |  |  |  |
| Low maximum temperature | <-1 SD vs. -1 – 1 SD | Natural Indirect Effect | -0.01 | -0.06 – 0.04 | 0.604 | 0.00 | -0.04 – 0.05 | 0.865 |
|  | <-1 SD vs. -1 – 1 SD | Natural Direct Effect | -0.73 | -1.33 - -0.13 | 0.016* | -0.79 | -1.39 - -0.18 | 0.011* |
|  | <-1 SD vs. -1 – 1 SD | Total Effect | -0.74 | -1.35 - -0.14 | 0.016* | -0.78 | -1.40 - -0.16 | 0.013* |
|  |  |  |  |  |  |  |  |  |
| Low maximum absolute humidity | <-1 SD vs. -1 – 1 SD | Natural Indirect Effect | 0.00 | -0.13 – 0.06 | 0.883 | 0.02 | -0.03 – 0.07 | 0.466 |
|  | <-1 SD vs. -1 – 1 SD | Natural Direct Effect | -1.00 | -1.66 – -0.34 | 0.003* | -1.06 | -1.73 - -0.40 | 0.002* |
|  | <-1 SD vs. -1 – 1 SD | Total Effect | -1.00 | -1.67 - -0.32 | 0.004* | -1.04 | -1.72 - -0.36 | 0.003* |
| **Fall** |  |  |  |  |  |  |  |  |
| High solar radiation | >0 SD vs. -1.5 – 0 SD | Natural Indirect Effect | -0.23 | -0.47 – 0.02 | 0.069 | -0.26 | -0.51 - -0.00 | 0.047* |
|  | >0 SD vs. -1.5 – 0 SD | Natural Direct Effect | -0.97 | -1.88 - -0.06 | 0.036* | -0.84 | -1.81 – 0.13 | 0.089 |
|  | >0 SD vs. -1.5 – 0 SD | Total Effect | -1.20 | -2.00 - -0.39 | 0.004* | -1.10 | -1.95 - -0.25 | 0.012* |

β = Beta coefficient

CI = Confident Interval

***** p-value < 0.05

^a^ Seasonal weather conditions were categorized into three groups by examining Lowess plots between the weather variable and both the mediator (time at home) and outcome (12-day lagged hospital admissions) in this analysis. Linear regression analyses compared the association of “high” and “low” weather categories (versus the mid-range) on both the mediator and outcome. Those seasonal weather conditions were significantly associated with both are included in this table.
